# Supplementary material for: Theta-Nested Gamma Oscillations in Next Generation Neural Mass Models
Source: Front Comput Neurosci. 2020 May 28;14:47. doi: 10.3389/fncom.2020.00047 (PMC7270590; doi:10.3389/fncom.2020.00047)
Supplement: Supplementary file 1 [file Presentation_1.pdf]

# Theta-nested gamma oscillations in next generation neural mass models

**Marco Segneri**[1], **Hongjie Bi**[1,2], **Simona Olmi**[3,4], **and Alessandro Torcini**[1,4,*]

[1] *Laboratoire de Physique Théorique et Modélisation, Université de Cergy-Pontoise,CNRS, UMR 8089, 95302 Cergy-Pontoise cedex, France*
[2] *Okinawa Institute of Science and Technology, Onna-son, Okinawa 904-0495, Japan*
[3] *Inria Sophia Antipolis Méditerranée Research Centre, 2004 Route des Lucioles, 06902 Valbonne, France*
[4] *CNR - Consiglio Nazionale delle Ricerche - Istituto dei Sistemi Complessi, via Madonna del Piano 10, 50019 Sesto Fiorentino, Italy*

Correspondence*:
Alessandro Torcini
alessandro.torcini@u-cergy.fr

## APPENDIX A: PING SET-UP: SUB-CRITICAL HOPF

In the PING set-up with only recurrent excitation (i.e. with $J^{(ee)} \neq 0$, $J^{(ii)} = 0$ and $J^{(ie)} = J^{(ei)} \neq 0$), it is possible to observe the emergence of COs also via a sub-critical Hopf bifurcation, by using $H^{(e)}$ as control parameter, as shown in Fig. 1 (a). This is due to the nature of the Hopf bifurcation that can be modified by simply varying the value of $H^{(i)}$. In this case we observe three regimes: an asynchronous one for $H^{(e)} < H^{(e)}_{SN}$; an oscillatory one for $H^{(e)} > H^{(e)}_c$ and a bistable one in the range $[H^{(e)}_{SN} : H^{(e)}_c]$. The frequency of the COs $\nu^{(e)}$ is always in the $\gamma$-range with a minimal value $\simeq 36$ Hz achievable at the Hopf bifurcation, see the inset of Fig. 1 (a).

If we consider the unforced system with $H^{(e)} < H^{(e)}_{SN}$ and we apply a $\theta$-forcing, we observe PAC oscillations. However when considering $v^{(e)}$, the COs are now asymmetric with respect to the maximum of the stimulation current $I^{(e)} = I_\theta(t)$ (see Fig. 1 (b)). This effect is even more pronounced by observing the wavelet spectrogram reported in Fig. 1 (c), where a clear PFC is also observable. The asymmetry in the onset of the gamma oscillations is clearly visible in the continuous wavelet transform obtained from the experimental data and reported in Fig. 4G in (Butler et al., 2016). This asymmetry can be explained in an adiabatic framework by considering the corresponding bifurcation diagram shown in Fig. 1 (a). Indeed for the sub-critical Hopf, the COs will emerge for $I_\theta > [H^{(e)}_c - H^{(e)}]$, but they will disappear for a different value of the forcing, namely $I_\theta < [H^{(e)}_{SN} - H^{(e)}]$. Instead, for a super-critical, Hopf the emergence and disappearence of the oscillations will occur at the same forcing amplitude, namely $I_\theta = [H^{(e)}_c - H^{(e)}]$.

## APPENDIX B: DIFFERENT PING SET-UPS

In the main text we have considered a unique configuration giving rise to COs via the PING mechanism: namely, two cross coupled inhibitory and excitatory populations with recurrent excitation and no recurrent inhibition (i.e. $J^{(ee)} \neq 0$ and $J^{(ii)} = 0$). However, other network configurations can give rise to PING induced oscillatory regimes. In particular, we have observed such oscillations with only cross-couplings in the absence of recurrent excitation and inhibition (i.e. $J^{(ee)} = J^{(ii)} = 0$), as well as in the presence

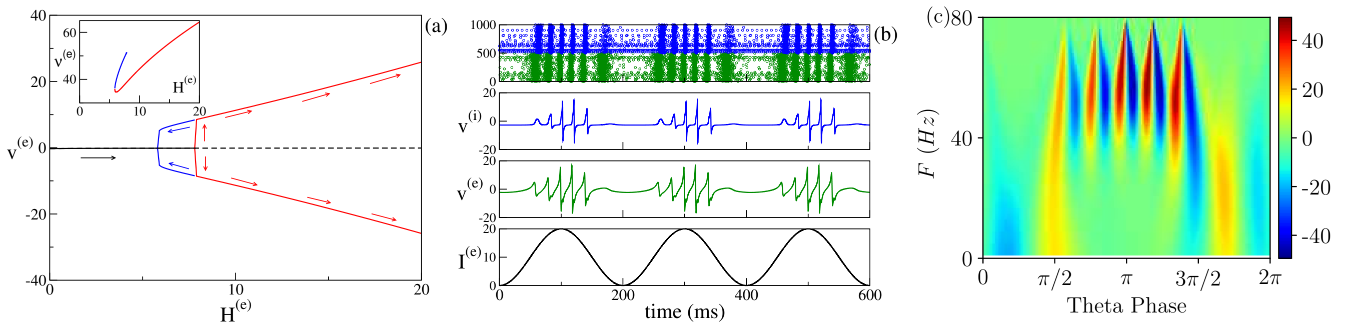


**Figure 1. (PING set-up: subcritical Hopf)** (a) Bifurcation diagram of the neural mass model of the average membrane potential $v^{(e)}$ as a function of $H^{(e)}$. The black continuous (dashed) line identifies the stable (unstable) fixed point. The red lines denote the maxima and minima of the limit cycles. The subcritical Hopf bifurcation occurs at $H_c^{(e)} \simeq 7.8$ while the saddle-node of limit cycles occurs at $H_{SN}^{(e)} = 5.8$. In the inset the COs' frequency $\nu^{(e)}$ is displayed as a function of $H^{(e)}$. (b) From top to bottom: raster plot where green (blue) dots refer to excitatory (inhibitory) neurons in a network of 10000 neurons; average membrane potentials $v^{(i)}$ and $v^{(e)}$ as obtained by the evolution of the neural mass models and forcing current $I^{(e)}$ for $H^{(e)} = -5 < H_{SN}^{(e)}$ and $\nu_\theta = 5$ Hz. (c) Continuous wavelet transform over a single $\theta$ cycle for $v^{(e)}$ with system setting as in (b). The remaining system parameters are $J^{(ee)} = 8$, $J^{(ii)} = 0$, $J^{(ie)} = J^{(ei)} = 10$, $H^{(i)} = -8.0$ and the size of the excitatory (inhibitory) network is $N^{(e)} = 5000$ ($N^{(i)}$ $= 5000$) of recurrent inhibition only (i.e. $J^{(ee)} = 0$ and $J^{(ii)} \neq 0$). In the following we refer to the former configuration as $\text{PING}_0$ set-up, while the latter configuration with recurrent inhibition is identified as $\text{PING}_I$ set-up. In both configurations the neural mass reproduces the emergence of $\gamma$ oscillations via a super-critical Hopf bifurcation for increasing values of $H^{(e)}$, as shown in Figs. 2 (a) and (b). Indeed the frequencies of the COs are in the range $[26 : 63.5]$ Hz ($[29.1 : 53.9]$ Hz) for $\text{PING}_0$ ($\text{PING}_I$) set-up. In both configurations the corresponding bifurcation, as a function of the parameter $H^{(i)}$, is sub-critical and COs disappear for sufficiently positive values of $H^{(i)}$, analogously to what is reported in the main text for the PING set-up with only recurrent excitation. It should be stressed that the standard Wilson-Cowan neural mass model gives rise to COs only in the presence of a recurrent excitation (Wilson and Cowan, 1972), thus being unable to reproduce the spiking network dynamics (Dumont and Gutkin, 2019).

In the presence of an external $\theta$-forcing with $\nu_\theta = 5$ Hz, we clearly observe $\theta$-nested $\gamma$-oscillations, as shown in the raster plots reported in Figs. 2 (top rows of panels (b) and (d)). These oscillations are phase amplitude modulated from the forcing, as it results to be evident from the shape of the mean membrane potentials $V^{(e)}$ and $V^{(i)}$ reported in the middle rows of Figs. 2 (c) and (d).

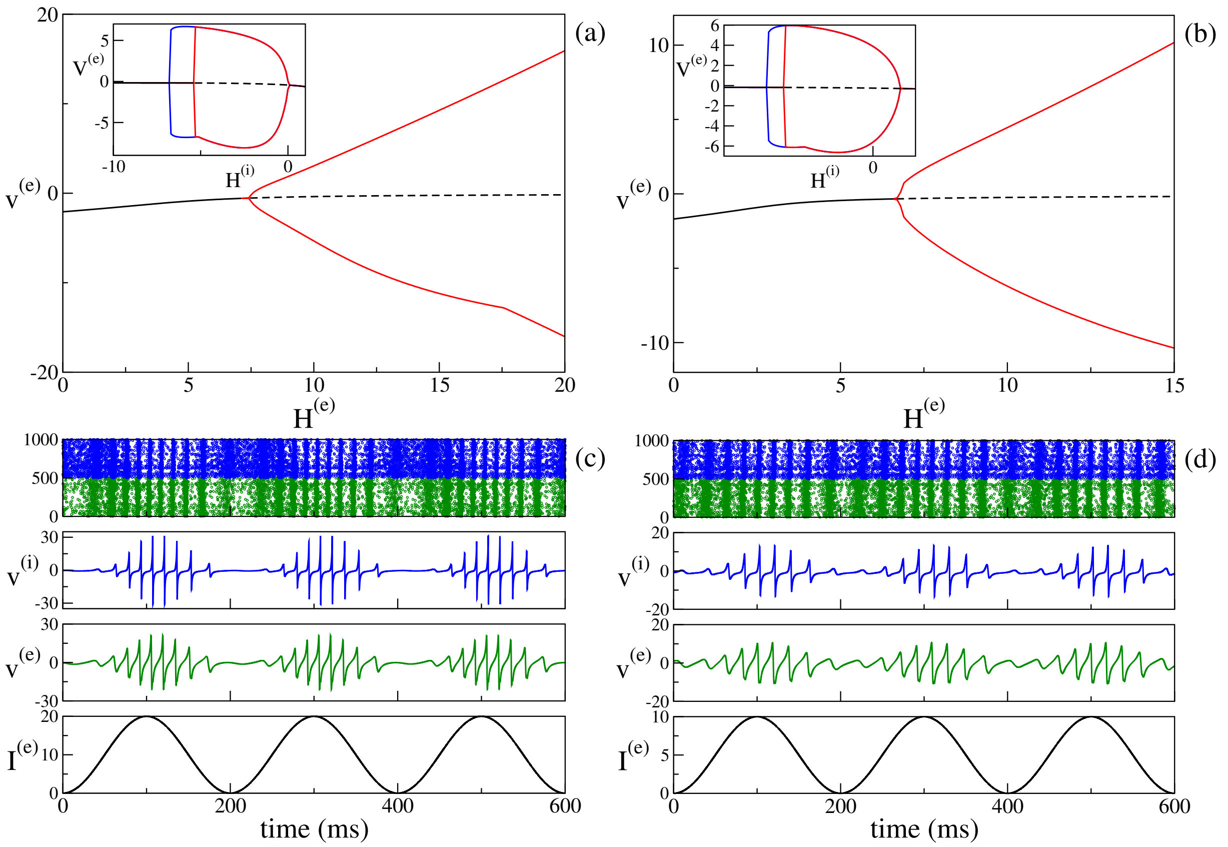


**Figure 2.** (**Different PING set-ups**) Bifurcation diagram for the neural mass model versus $H^{(e)}$, for the PING$_0$ (a) and PING$_I$ (b) set-ups for $H^{(i)} = -0.5$. The corresponding insets show the bifurcation diagrams as a function of $H^{(i)}$, for $H^{(e)} = 10$. $\theta$-nested $\gamma$ oscillations emerging in the PING$_0$ (c) and PING$_I$ (d) configurations for $I_0 = 20$ and $\nu_\theta = 5$ Hz. From top to bottom the raster plot where green (blue) dots refer to excitatory (inhibitory) neurons in a network of 10000 neurons; the average membrane potentials $v^{(i)}$ and $v^{(e)}$ as obtained by the evolution of the neural mass models and the forcing currents $I^{(e)}$. Parameters for the PING$_0$ set-up are $J_{ee} = J_{ii} = 0$, while for PING$_I$ are $J_{ee} = 0$ and $J_{ii} = 8$. In both cases $J_{ie} = J_{ei} = 10$ and $H^{(i)} = -0.5$. In the corresponding insets we set $H^{(e)} = 10$. The size of the excitatory (inhibitory) network shown in panels (c), (d) is $N^{(e)} = 5000$ ($N^{(i)} = 5000$).

## REFERENCES

Butler JL, Mendonça PR, Robinson HP, Paulsen O. Intrinsic cornu ammonis area 1 theta-nested gamma oscillations induced by optogenetic theta frequency stimulation. *Journal of Neuroscience* **36** (2016) 4155–4169.

Wilson HR, Cowan JD. Excitatory and inhibitory interactions in localized populations of model neurons. *Biophysical journal* **12** (1972) 1–24.

Dumont G, Gutkin B. Macroscopic phase resetting-curves determine oscillatory coherence and signal transfer in inter-coupled neural circuits. *PLoS computational biology* **15** (2019) e1007019.
